# Identifying S3 and S2 as Key Pain-Sensitive Targets in High-Frequency Ultrasound Therapy for Sacroiliitis

**DOI:** 10.3390/jcm14238314

**Published:** 2025-11-22

**Authors:** Itay Goor-Aryeh, Paz Kelmer, Ruth Gur, Tal Harel, Roee Sheinfeld, Oded Jacobi, Lior Ungar

**Affiliations:** 1 Pain Center, Sheba Medical Center, Tel Hashomer, Ramat Gan 52621, Israel; 2 Department of Neurosurgery, Sheba Medical Center, Tel Hashomer, Ramat Gan 52621, Israel

**Keywords:** sacroiliitis, high-frequency ultrasound, sacral nerve branches

## Abstract

**Background/Objectives:** Sacroiliitis is a painful inflammatory disorder of the sacroiliac joint, estimated to account for up to 25% of chronic low back pain. Treatment options are often limited, and many patients continue to experience symptoms despite conservative or interventional management. High-Intensity Focused ultrasound (HIFU) has emerged as a novel noninvasive neuromodulation technique. However, the contribution of individual lumbosacral nerve branches (L5–S3) to pain generation during such interventions remains unclear. This study aimed to characterize the distribution of pain-related interruptions during HIFU procedures, with a particular focus on identifying the most pain-sensitive targets. **Methods:** Eight patients with clinically confirmed sacroiliitis underwent HIFU ablation targeting the L5–S3 branches. Procedural data, including the total number of sonications and interruptions due to pain, were prospectively recorded. Statistical analyses were performed using chi-square tests, including overall distribution testing, pairwise branch comparisons, and an aggregated comparison of S3 versus all other branches combined. Effect sizes were calculated using Cohen’s w. **Results:** Across 243 sonications, 162 interruptions (66.7%) occurred due to pain. Interruptions were unevenly distributed: 81% occurred at S2 and S3, with S3 alone accounting for 42%. S3 showed significantly more interruptions than L5 (*p* = 0.0022), S1 (*p* = 0.0150), S2 (*p* = 0.0055), and all other branches combined (*p* < 0.001; w = 0.58, large effect). S2 also demonstrated greater sensitivity than L5 (*p* = 0.003) and S1 (*p* = 0.001). Subdivision analysis revealed uniformly high sensitivity across S3, whereas S1 and S2 displayed heterogeneous patterns. **Conclusions:** HIFU stimulation revealed disproportionate pain sensitivity in sacral branches, with S3—and to a lesser extent S2—emerging as dominant contributors. These findings provide new insight into sacroiliitis pathophysiology and suggest prioritization of S3 in targeted interventional management.

## 1. Introduction

The sacroiliac (SI) joint is a major synovial articulation that plays a critical role in transferring load between the spine and pelvis. Inflammation of this joint, or sacroiliitis, is recognized as a common cause of buttock and lower back pain, contributing to an estimated 10–25% of cases of chronic low back pain, with prevalence increasing with age and after lumbar fusion procedures [1,2,3,4]. Symptoms most frequently localize to the ipsilateral buttock (94%) and midline lumbar region (74%) [5,6], but may also radiate to the thigh or hip, complicating diagnosis and management [7]. Emphasizing the diagnostic challenges due to overlapping symptom patterns with lumbar spine, hip, and gluteal pathologies. Differential diagnoses include hip osteoarthritis, muscular imbalance, and leg-length discrepancies [8]. Other important differential diagnoses include greater trochanteric pain syndrome (GTPS), hip osteoarthritis, piriformis syndrome, and referred pain from lumbar facet arthropathy. The etiology of sacroiliitis includes both degenerative and inflammatory causes. Rheumatologic conditions such as axial spondyloarthritis, ankylosing spondylitis, and psoriatic arthritis are among the most frequent systemic contributors and should be considered in the diagnostic workup [9,10]. Pathological findings vary widely, ranging from synovial inflammation to subchondral bone changes, though some patients exhibit no histological abnormalities [11,12].

While sacroiliitis is clinically well recognized, the underlying neural mechanisms that generate pain remain incompletely understood. Pain may arise from both intra-articular structures and posterior ligamentous tissues [13,14]. The sensory innervation of the SI joint is complex, involving contributions from the dorsal rami of the L5, S1, S2, and S3 nerve roots. However, the relative importance of each branch in mediating nociception has not been clearly defined. Limited anatomical and clinical data suggest variability in how these nerve roots contribute to pain distribution, which may explain the heterogeneity of symptoms observed in patients with sacroiliitis.

Conservative management—such as physical therapy, nonsteroidal anti-inflammatory drugs, and image-guided injections—remains first-line, while radiofrequency ablation and, rarely, fusion surgery are considered for refractory cases [15,16,17]. Minimally invasive interventions, such as radiofrequency (RF) and pulsed radiofrequency (PRF) ablation, have demonstrated clinical benefit in managing sacroiliac pain through selective denervation of the posterior sacral network [15,16,17]. However, these approaches are limited by variability in lesion geometry, invasiveness, and post-procedure discomfort. In recent years, non-invasive neuromodulation technologies such as high-intensity focused ultrasound (HIFU) have emerged as potential therapeutic options. Unlike radiofrequency ablation, HIFU allows for noninvasive, image-guided targeting of neural structures [18,19,20,21,22]. However, the relationship between specific sacral nerve branches and procedural pain responses during such treatments remains poorly characterized.

The sensory innervation of the sacroiliac joint is both complex and incompletely defined. Anatomical and cadaveric studies have shown that the joint receives contributions from the dorsal rami of L5 as well as the lateral branches of S1–S3, with variable overlap between individuals [23]. Fortin and colleagues demonstrated that provocation of the sacroiliac joint commonly produces buttock and posterior thigh pain, implicating sacral dorsal rami in symptom generation [24]. More recent cadaveric dissections confirmed that the posterior sacroiliac ligaments are richly innervated by the lateral branches of S1–S3, providing a plausible substrate for nociceptive signaling [25].

Despite these observations, the relative importance of individual nerve roots in transmitting pain from the sacroiliac joint remains poorly understood. Clinical data directly comparing sensitivity across L5–S3 branches are scarce, leaving uncertainty regarding which divisions are most critical in sacroiliitis pathophysiology. Clarifying this distribution is essential, as it may explain the heterogeneous symptom patterns observed in patients and support the development of more precise interventional strategies.

The present study addresses this gap by prospectively evaluating pain-related interruptions during high-intensity focused ultrasound treatment of sacroiliitis. This was a uniquely feasible occurrence, as HIFU treatment of sacroiliitis is more painful compared to HIFU ablation of the lumbar medial branch which is usually uninterrupted. Also, as newer treatment protocols extend energy delivery time and thereby reduce intraprocedural pain, future procedures are unlikely to generate such data. By analyzing patterns across different lumbosacral branches, this work provides preliminary evidence of differential nerve root sensitivity and identifies potential therapeutic targets.

Therefore, the aim of this study was to identify which lumbosacral branches are most sensitive to pain during high-frequency focused ultrasound treatment for sacroiliitis, providing neurophysiological insight and guiding future interventional targeting.

## 2. Materials and Methods

### 2.1. Participants

Eight patients (five women, three men), 55 years old on average, with a mean BMI of 26, with chronic sacroiliac joint (SIJ) pain, were prospectively enrolled. Most patients were healthy with no Major Comorbidities except one patient who suffered from hypertension. All participants met the diagnostic criteria of the International Association for the Study of Pain (IASP) for SIJ-related low back pain [26]. The average pain duration was 3.2 years

Inclusion criteria consisted of adults aged 18–75 years with a diagnosis of sacroiliac joint pain confirmed by ≥75% pain relief following diagnostic intra-articular injection, concordant clinical presentation, and radiographic or MRI evidence of SIJ pathology. Exclusion criteria included active systemic infection, prior sacral surgery, uncontrolled coagulopathy, pregnancy, or concurrent spinal/hip pathology capable of confounding pain origin. Local and national institutional review board (IRB) and regulatory approvals (by the Sheba Medical Center Institutional Review Board, 9283-22-SMC, 13 March 2022) were obtained before study initiation, and written informed consent was secured from all patients prior to treatment.

### 2.2. Procedure

All interventions were conducted in the Interventional Pain Department at Sheba Medical Center. Patients were positioned prone on a C-arm table (OakWorks Medical, New Freedom, PA, USA). Imaging guidance was provided using a PHILIPS ZENITION 70 GE 12-inch C-arm (Boston, MA, USA).

Targeting and ablation were performed with the Neurolyser XR system (FUSMobile Inc., Alpharetta, GA, USA), a fluoroscopy-guided high-frequency ultrasound (HIFU) platform. The device couples an ultrasonic transducer with X-ray guidance, enabling anatomical localization and precise energy delivery to neural targets adjacent to bony structures. Acoustic coupling was achieved using a disposable gel pad, which facilitated ultrasound energy transfer into the body while preventing energy loss. A lateral fluoroscopic image of the pelvis was first obtained to assess the skin-to-bone distance along the posterior sacral surface. This measurement was used to confirm that the region containing the targeted sacral nerve branches lay within the NeurolyserXR therapeutic envelope, which was digitally superimposed on the live X-ray image. Once proper alignment was verified, the procedure commenced. For each treatment site, an anteroposterior (AP) image was acquired, and the transducer was positioned under real-time software guidance to ensure precise spatial orientation. Target localization was further validated using a secondary image acquisition of the system’s integrated X-ray aim, superimposed on the patient’s anatomy, allowing fine adjustments to optimize targeting accuracy before energy delivery. Although the NeurolyserXR platform integrates an ultrasound-based therapeutic transducer [27], targeting and confirmation were performed using real-time fluoroscopy (X-ray) overlay to ensure precise alignment of the focal zone with the posterior sacral surface. This dual-modality system minimizes targeting error and enhances safety compared to conventional free-hand ultrasound guidance.

For each target, an initial verification sonication (non-ablative, 300 J for 20 s) was delivered to ensure proper positioning and to exclude mistargeting. Once confirmed, therapeutic sonications were administered at ablative settings (1000 J for 50 s). No sedation was administered, allowing continuous patient feedback. During sonication, either the patient or clinical staff could terminate energy delivery if pain or motor/sensory responses suggestive of mistargeting occurred. In cases where energy delivery was interrupted due to pain alone, treatment was paused for <30 s and then resumed until ablation was complete. This sequence was repeated until all planned targets were addressed. Pain-related interruptions were defined as voluntary termination of sonication in response to acute pain rated ≥6/10 on a numerical scale. For reproducibility, interruption frequencies were normalized to the total acoustic energy delivered per branch (interruptions/Joule), allowing intersubject comparison independent of procedural duration or output.

### 2.3. Data Collection

For each patient, all sonications and interruptions were documented in an excel sheet, including the anatomical target (L5–S3, subdivided into inferior, middle, and superior branches where applicable), total number of energy deliveries, and number of interruptions due to pain.

### 2.4. Statistical Analysis

Comparisons of interruption frequencies across the main branches (L5, S1, S2, S3) were performed using chi-square goodness-of-fit tests. Pairwise comparisons (S3 vs. L5, S3 vs. S1, S3 vs. S2) and the aggregated analysis of S3 versus all other branches combined were conducted using chi-square tests of independence on 2 × 2 contingency tables. Effect sizes were calculated using Cohen’s *w*. Statistical significance was defined as *p* < 0.05. Given the exploratory nature of this first-in-human feasibility study, no formal power calculation was performed. The sample size of eight was determined pragmatically to establish procedural feasibility and gather initial neurophysiological data for future hypothesis generation.

### 2.5. Use of AI Tools

During the preparation of this study, the authors used OpenAI’s ChatGPT (GPT-5.1 model) and Microsoft Copilot (GPT-5 model integrated into the Microsoft Word application, November 2025 release) to support image generation and writing-related editing. All outputs produced by these tools were thoroughly reviewed and revised by the authors. The authors take full responsibility for the accuracy and integrity of the content presented in this publication.

## 3. Results

Eight patients (five women, three men) underwent bilateral sonications with 243 sonications targeting lumbosacral branches (L5–S3). Across all procedures, 162 interruptions (66.7%) were recorded due to patient-reported pain. Across patients, the mean number of sonications per session was 30.4 ± 8.7 (range 22–46). Mean delivery of energy per site was 870 ± 210 J. Interruption rates were consistent across patients (mean 66.7%, SD 11.4%).

Interruptions were unevenly distributed across the main branches. S3 accounted for 68 interruptions (42.0%), S2 for 63 (38.9%), S1 for 21 (13.0%), and L5 for 10 (6.2%). The difference across main branches was highly significant (χ^2^(3) = 63.5, *p* < 0.001; Cohen’s *w* = 0.63, large effect).

**Figure 1:**
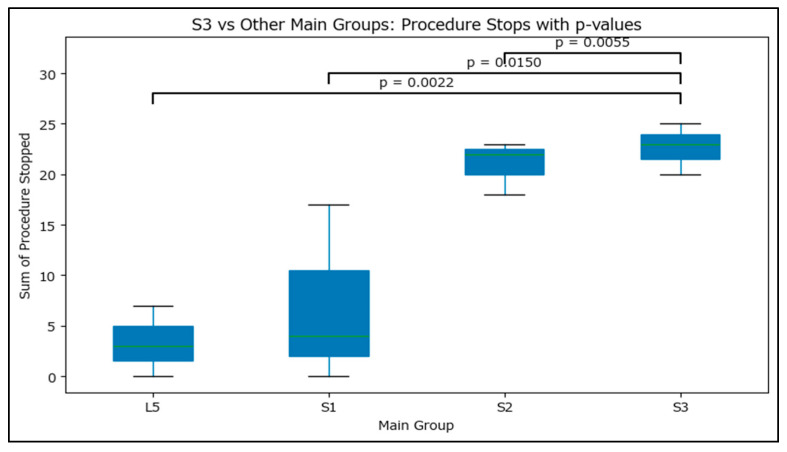
Boxplot illustrating the distribution of interruption counts during high-intensity focused ultrasound (HIFU) sonications at the L5–S3 branches. The S3 branch exhibited a significantly higher rate of procedural interruptions compared with all other levels.

Pairwise analyses confirmed that S3 produced significantly more interruptions than L5 (*p* = 0.0022), S1 (*p* = 0.0150), and S2 (*p* = 0.0055) (Figure 1). An aggregated comparison further demonstrated that S3 had significantly more interruptions than all other branches combined (*p* < 0.001; Cohen’s *w* = 0.58, large effect). Additional pairwise testing among the non-S3 branches revealed no difference between L5 and S1 (*p* = 0.95), but significantly more interruptions at S2 compared with both L5 (*p* = 0.003) and S1 (*p* = 0.001).

**Figure 2:**
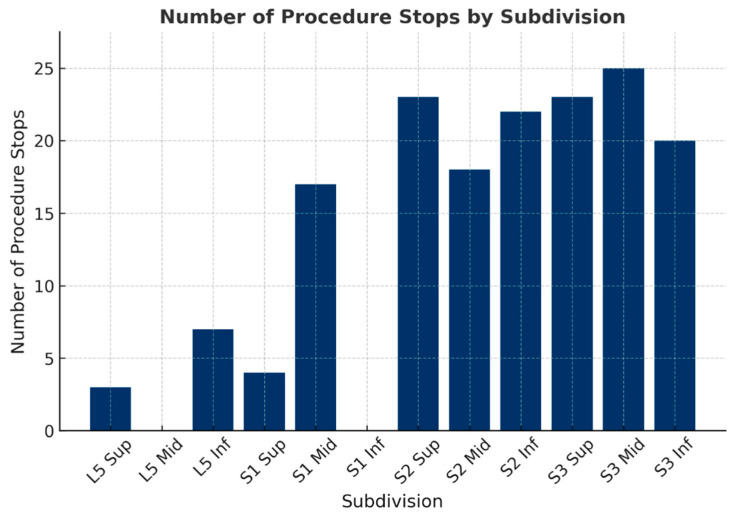
Interruption counts varied among subdivisions, with clustering at S1-mid and uniformly high sensitivity across all S3 levels. These findings suggest localized nociceptive responses in S1 and S2, and consistent sensitivity throughout S3.

Subdivision-level analysis revealed distinct patterns across the sacral branches (Figure 2). Within S3, interruption rates were uniformly high across all subdivisions—superior (82.1%), middle (86.2%), and inferior (71.4%)—with no significant differences among them (χ^2^(2) = 0.56, *p* = 0.76), indicating a consistent pain sensitivity profile throughout the nerve’s trajectory. In contrast, S1 and S2 demonstrated heterogeneous distributions. At S1, interruptions clustered prominently at the middle subdivision (χ^2^(2) = 22.6, *p* < 0.001), whereas S2 exhibited reduced sensitivity at its superior and high subdivisions compared with the mid and inferior levels (*p* < 0.05). These findings suggest that while S3 functions as a uniformly sensitive pain mediator, the distribution of nociceptive responses in S1 and S2 may be anatomically localized to specific branches.

**Figure 3:**
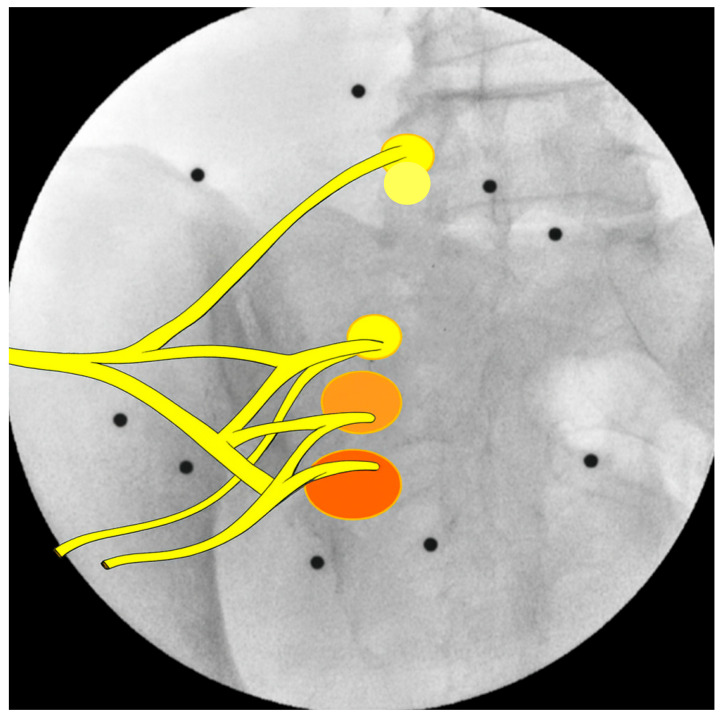
Color-graded overlays demonstrate a caudal increase in nociceptive responsiveness: with S3 branch having the highest sensitivity (deep orange), followed by S2, S1, and L5, (progressively lighter) indicating a gradient of nociceptive responsiveness increasing caudally.

When interruption frequency was normalized to total delivered energy, a distinct gradient of nociceptive responsiveness emerged across the lumbosacral branches (Table 1). The S3 branch exhibited the highest sensitivity index, followed by S2, whereas S1 and L5 demonstrated substantially lower ratios of interruptions per energy unit. This graded pattern highlights a progressive decline in pain sensitivity from the lower sacral to the upper lumbar levels, suggesting a functional hierarchy in nociceptive activation within the sacral plexus. (Figure 3).

Together, these findings consistently identify S3 as the most pain-sensitive target during HIFU treatment of sacroiliitis, with S2 also demonstrating increased sensitivity relative to L5 and S1.

## 4. Discussion

This prospective evaluation of eight patients with sacroiliitis undergoing high-frequency ultrasound (HIFU) ablation demonstrated that treatment interruptions due to pain were strongly concentrated at the sacral nerve roots, particularly at S3. Among the 162 interruptions recorded, more than 80% occurred at S2 and S3, with S3 alone accounting for the highest proportion. Statistical analysis confirmed that interruptions were significantly more frequent at S3 compared to each of the other lumbosacral branches, and when analyzed against all branches combined.

Interestingly, pairwise comparisons among non-S3 branches revealed that S2 also exhibited greater sensitivity than both L5 and S1, while no difference was detected between L5 and S1. This suggests that both S2 and S3 play a substantial role in the nociceptive pathways of sacroiliac pain, though S3 remains the most consistently pain-sensitive root. These findings align with anatomical evidence indicating that sacral lateral branches, especially S2 and S3, provide major sensory innervation to the sacroiliac joint and its supporting ligaments [23,24,25].

The identification of S3, and to a lesser extent S2, as key contributors to pain during HIFU treatment has important clinical implications. Neuroablative procedures such as radiofrequency denervation typically target multiple sacral branches; however, these results suggest that prioritizing S2 and S3, particularly S3, may enhance therapeutic precision and efficacy. Conversely, interventions directed at L5 and S1 may yield limited benefit, as these branches accounted for only a small proportion of pain-related interruptions.

At the subdivision level, S3 displayed a strikingly uniform pattern of pain sensitivity, with comparable interruption rates across superior, middle, and inferior branches. This suggests a diffuse nociceptive response throughout the S3 distribution, consistent with its broad sensory supply to the sacroiliac ligaments and posterior pelvic structures. In contrast, S1 and S2 showed more variable profiles, with interruptions clustering around the mid-level branches. These differences may reflect the variable course and depth of the lateral sacral branches, as well as the differential accessibility of terminal fibers to ultrasound energy. Together, these findings reinforce the functional prominence of S3 while revealing potentially distinct mechanosensory roles for the more cranial sacral branches.

In comparison with existing neuromodulation techniques, conventional interventions for sacroiliac pain, including sacral nerve stimulation (SNS) and radiofrequency ablation (RFA), commonly target the S3 nerve root due to its dominant sensory role. While SNS offers neuromodulatory benefits, it requires implanted hardware and carries procedural risks; RFA, though effective, may result in nonselective lesioning and inconsistent targeting [28,29]. In contrast, high-frequency focused ultrasound (HIFU) enables noninvasive, image-guided, and precise focal energy delivery to neural targets. The consistent pain sensitivity observed at S3 in this study supports its use as a potential HIFU target, and future comparative trials will be needed to determine whether this technique yields superior efficacy or durability compared with existing neuromodulation methods.

Strengths of this study include its prospective design, systematic recording of procedural events, and use of HIFU to provoke consistent nociceptive responses at anatomically defined sites. However, limitations must be acknowledged. The small cohort size may restrict generalizability, and interruptions during sonication were used as surrogate markers of sensitivity rather than direct measures of pain perception. In addition, the impact of the specific gel pad used, as well as skin-to-bone depth, was neglected due to the small sample size. Interindividual variability in sacral innervation may also influence results, and larger studies are required to validate these findings and assess their clinical utility. Nevertheless, the absence of a control group represents an additional limitation. Future large-scale, multicenter, randomized controlled trials will be essential to validate these findings and to further optimize treatment parameters.

The present study fills an important knowledge gap by prospectively characterizing pain-related interruptions during high-intensity focused ultrasound (HIFU) treatment for sacroiliitis. This unique observation window was possible because HIFU applied to the sacroiliac region is typically more painful and less tolerable than its use for lumbar medial branch ablation, which is generally uninterrupted. Moreover, as evolving treatment protocols increasingly emphasize prolonged, lower-intensity energy delivery to minimize intraprocedural discomfort, comparable data are unlikely to be reproduced in future clinical settings. By systematically analyzing interruption patterns across lumbosacral nerve branches, this study provides preliminary evidence of differential nerve root sensitivity to ultrasonic energy. When normalized for total energy delivered, S3 and, to a lesser extent, S2 exhibited markedly higher interruption rates per energy unit, suggesting greater nociceptive responsiveness. These findings offer novel insight into the functional topography of sacral nerve roots and highlight S3 as a potential therapeutic focus for refining targeted neuromodulation strategies in sacroiliitis. Future research could integrate real-time EMG or fMRI feedback to quantify central processing associated with peripheral nerve stimulation. Larger cohorts could validate whether the S3-dominant sensitivity observed here correlates with clinical pain localization patterns or therapeutic outcomes following targeted denervation.

## 5. Conclusions

This study highlights S3 as the most pain-sensitive target during HIFU treatment of sacroiliitis, with S2 also showing significant but less dominant involvement. Together, these findings underscore the importance of sacral nerve branches, particularly S3, in the pathophysiology of sacroiliac pain and provide a rationale for refining interventional strategies to focus on these targets. Future research should validate these findings in larger, multicenter cohorts and explore the integration of image-guided neuromodulation or real-time pain mapping to further optimize target selection and therapeutic precision in sacroiliac interventions.

## Figures and Tables

**Table 1:** Relative sensitivity of lumbosacral branches based on interruption-to-energy ratio.

**Branch**	**Stopped/E**	**Interpretation**
S3	~0.0045	Highest sensitivity—strong reaction to small energy input
S2	~0.0030	Moderately high sensitivity
S1	~0.0018	Lower sensitivity
L5	~0.0003	Least sensitive

## Data Availability

Data is available by contacting the corresponding author upon reasonable request.
